# Evaluation of Methylation Biomarkers for Detection of Circulating Tumor DNA and Application to Colorectal Cancer

**DOI:** 10.3390/genes7120125

**Published:** 2016-12-15

**Authors:** Susan M. Mitchell, Thu Ho, Glenn S. Brown, Rohan T. Baker, Melissa L. Thomas, Aidan McEvoy, Zheng-Zhou Xu, Jason P. Ross, Trevor J. Lockett, Graeme P. Young, Lawrence C. LaPointe, Susanne K. Pedersen, Peter L. Molloy

**Affiliations:** 1 CSIRO Food and Nutrition, P.O. Box 52, North Ryde, NSW 1670, Australia; smitch7@gmail.com (S.M.M.); thu.ho@csiro.au (T.H.); glenn.brown@csiro.au (G.S.B.); zheng-zhou.xu@csiroalumni.org.au (Z.-Z.X.); jason.ross@csiro.au (J.P.R.); trevor.lockett@csiro.au (T.J.L.); 2 Clinical Genomics Pty Ltd., North Ryde, NSW 2113, Australia; rohan@geneticsignatures.com (R.T.B.); thomas_mel@hotmail.com (M.L.T.); aidan.mcevoy@clinicalgenomics.com (A.M.); larry@clinicalgenomics.com (L.C.L.); susanne.pedersen@clinicalgenomics.com (S.K.P.); 3 Flinders Centre for Innovation in Cancer, Flinders University of South Australia, GPO Box 2100, Adelaide, SA 5001, Australia; graeme.young@flinders.edu.au

**Keywords:** hypermethylation, biomarkers, colorectal cancer, plasma, *BCAT1*, *GRASP*, *IKZF1*, *IRF4 SDC2*, *SEPT9*, epigenetics, circulating tumor DNA, liquid biopsy

## Abstract

Solid tumors shed DNA into circulation, and there is growing evidence that the detection of circulating tumor DNA (ctDNA) has broad clinical utility, including monitoring of disease, prognosis, response to chemotherapy and tracking tumor heterogeneity. The appearance of ctDNA in the circulating cell-free DNA (ccfDNA) isolated from plasma or serum is commonly detected by identifying tumor-specific features such as insertions, deletions, mutations and/or aberrant methylation. Methylation is a normal cell regulatory event, and since the majority of ccfDNA is derived from white blood cells (WBC), it is important that tumour-specific DNA methylation markers show rare to no methylation events in WBC DNA. We have used a novel approach for assessment of low levels of DNA methylation in WBC DNA. DNA methylation in 29 previously identified regions (residing in 17 genes) was analyzed in WBC DNA and eight differentially-methylated regions (DMRs) were taken through to testing in clinical samples using methylation specific PCR assays. DMRs residing in four genes, *BCAT1*, *GRASP*, *IKZF1* and *IRF4*, exhibited low positivity, 3.5% to 7%, in the plasma of colonoscopy-confirmed healthy subjects, with the sensitivity for detection of ctDNA in colonoscopy-confirmed patients with colorectal cancer being 65%, 54.5%, 67.6% and 59% respectively.

## 1. Introduction

Tissue biopsies have long served as a source of biological material for pathology testing to aid physicians in diagnosis, prognosis and therapy selection. Recent research and commercialization efforts in the field of detecting circulating tumor DNA (ctDNA) in bodily fluids, i.e. “liquid biopsies”, particularly blood plasma, have demonstrated the clinical utility of detecting ctDNA as an aid for clinical management of cancer patients. The detection of ctDNA can be used for obtaining diagnostic, prognostic and theranostic information concerning cancer. The diagnostic utility of ctDNA primarily stems from the presence of somatic genomic alterations, such as in the *KRAS*, *BRAF* and *EGFR* genes, which are absent from DNA taken from matched normal cells and in the circulating cell-free DNA (ccfDNA) of healthy subjects [1,2,3,4,5,6].

Aberrant DNA methylation is a characteristic of most types of solid cancer with common hypermethylation events occurring more frequently than most mutations [7]. The specific patterns of DNA methylation differ between cancer types and in some cases can be used for classification of different sub-types [8]. For a number of cancers it has been possible to identify particular genes that are methylated with high frequency, e.g., *GSTP1* in prostate cancer [9,10], *SHOX2* in lung cancer [11], *SEPT9*, *NRDG4*, *RASSF1A*, *THBD*, *BCAT1* and *IKZF1* in colorectal cancer (CRC) [12,13,14,15,16]. These hypermethylation events are not confounded by the need to cover multiple and often large regions for possible mutations (such as, for example, *EGFR*, *APC*, *PIK3CA*). Hence there has been a growing interest in the application of assays for the detection of ctDNA by testing for specific methylated DNA biomarkers [17,18,19,20,21,22].

To transition DNA methylation markers validated in cancer tissue to blood-based assays, it is important to interrogate the frequency of methylation in the target gene in ccfDNA of healthy subjects. Bisulfite sequencing of DNA isolated from plasma has also been used recently for identification of commonly methylated sequences in healthy and diseased subjects [23,24]. The primary source of ccfDNA is white blood cell (WBC) DNA, contributing 80% in healthy subjects, with liver-derived DNA also contributing up to 10% [23]. We and others have previously used the level of methylation in WBC DNA as a selection criterion for ranking candidate markers [13,15,25,26,27].

We previously used a pipeline combining gene expression, targeted DNA methylation and genome-wide DNA methylation analyses to identify a panel of genes that are methylated in a high number of colorectal cancers [15]. Within this dataset we prioritized genes for further investigation based on other published literature as well as data available at that time from The Cancer Genome Atlas (TCGA) project. Here we describe an approach to identifying candidate biomarkers that are most promising for development of plasma assays, and evaluate them in pilot plasma samples from colorectal cancer cases and control subjects. Subsequently, two candidate biomarkers selected using this approach, *IKZF1* and *BCAT1* [28,29], have been evaluated for detection of ctDNA in larger patient cohorts.

## 2. Materials and Methods

### 2.1. DNA Samples

Clinical specimens were obtained with informed consent Southern Adelaide Health Service/Flinders University Human Research Ethics Committee Approval number 134/045 (First approved 2006, with amendment 19 January 2011) and trial registration ACTRN12611000318987 (approval, 16.03.2011). Buffy coat WBC DNA was obtained from nine male and nine female healthy donors under the age of 30, using the PAXgene blood DNA kit (QIAGEN, Hilden, Germany). Plasma samples were obtained from colonoscopy-confirmed patients from either Flinders Medical Centre (Adelaide, Australia) or sourced through Proteogenex Inc. (Culver City, CA, USA) as previously described in [28,30]. Clinical diagnosis was determined on the basis of colonoscopic findings and histological assessment. Cancers were staged according to American Joint Committee on Cancer (AJCC) Cancer staging 7th edition [31]. ccfDNA was isolated from 4 mL plasma using the QIAamp circulating nucleic acid kit (QIAGEN), and bisulphite-converted using an Epitect Fast bisulphite conversion kit (QIAGEN) as previously described [28].

### 2.2. MethylMiner Enrichment of Genomic DNA

Separate pools of male and female human genomic DNA were created by combining equal amounts of DNA from nine individuals. Samples of pooled DNA in a 300 µL volume of Tris-EDTA buffer (TE) were sonicated for 10 min in a Diagenode Bioruptor (Liege, Belgium) set on high. Sonication was performed for 20 cycles consisting of a 30s pulse followed by a 30 s rest. 1.8–3 µg of sonicated DNA (500 bp average size) was enriched for methylated fragments using the MethylMiner kit (Invitrogen, Carlsbad, CA, USA, Catalogue # ME10025) according to the manufacturer’s instructions and both bound and unbound fractions kept. The fractionated DNA was then subjected to bisulphite treatment using the EZ DNA Methylation-Gold Kit (Zymo Research, Irvine Ca, USA, Cat # D5006) according to the manufacturer’s instructions.

### 2.3. Real-time PCR Quantification

Bisulphite treated fractions were analyzed using real-time PCR assays designed for converted DNA sequences of each targeted gene region (Table S1). Equal aliquots of bound and unbound DNA were used in the reactions which were carried out in a 15 µL volume containing GoTaq Colourless Master Mix (Promega, Madison, Wi, USA, Cat # M5133), 3 mM MgCl_2_, 200 nM primer pairs, 1/100,000 SYBR Green dye at an annealing temperature ranging from 54 °C to 58 °C in a Light Cycler 480 II instrument (Roche Life Science, North Ryde, Australia). Melt curve analysis was performed on the amplicons generated as per manufacturer’s protocols.

### 2.4. Methylation Specific PCR Assays

Methylation-specific PCR assays (probe-based MethyLight assays or using SYBR Green detection) were run on ccfDNA samples isolated from patients as described above. Primer and probe sequences and PCR conditions are given in Table S2. For three assays using SYBR Green for amplicon detection, *PDX1*, *SDC2* and *IKZF1*, melt curves were used to confirm positive signals. All assays reliably detected the presence of target sequences at inputs of 20 pg or less of fully methylated genomic DNA. The number of positive assays in triplicate are shown in Table S3. A sample was designated positive if at least one PCR triplicate had a methylation signal above background, although a threshold cut (pg methylated DNA per mL plasma, averaged across the triplicates) was imposed for the *IRF4* assay (see Table S2). Detection of methylated *SEPT9* sequences was done using the described HeavyMethyl assay [12].

## 3. Results

Based on a previous panel of genes demonstrated to be methylated in a high fraction of colorectal cancer tissue specimens [15], and using TCGA CRC methylation profiles (cancer/normal profiles shown in Figure S1), we chose a set for more detailed evaluation as potential markers for blood-based assays for CRC detection. DNA methylation array data plots for genes are shown in Figure S1. The set comprised *BCAT1*, *EFEMP1*, *FGF5*, *FOXI2*, *GATA2*, *GRASP*, *HOXA2*, *HOXA5*, *IKZF1*, *IRF4*, *PDX1*, *SDC2*, *SLC6A15*, *SNCB, SOX21*, *ZNF471* and *ZSCAN18*.

### 3.1. Characterisation of Methylation in WBC DNA

**Figure 1:**
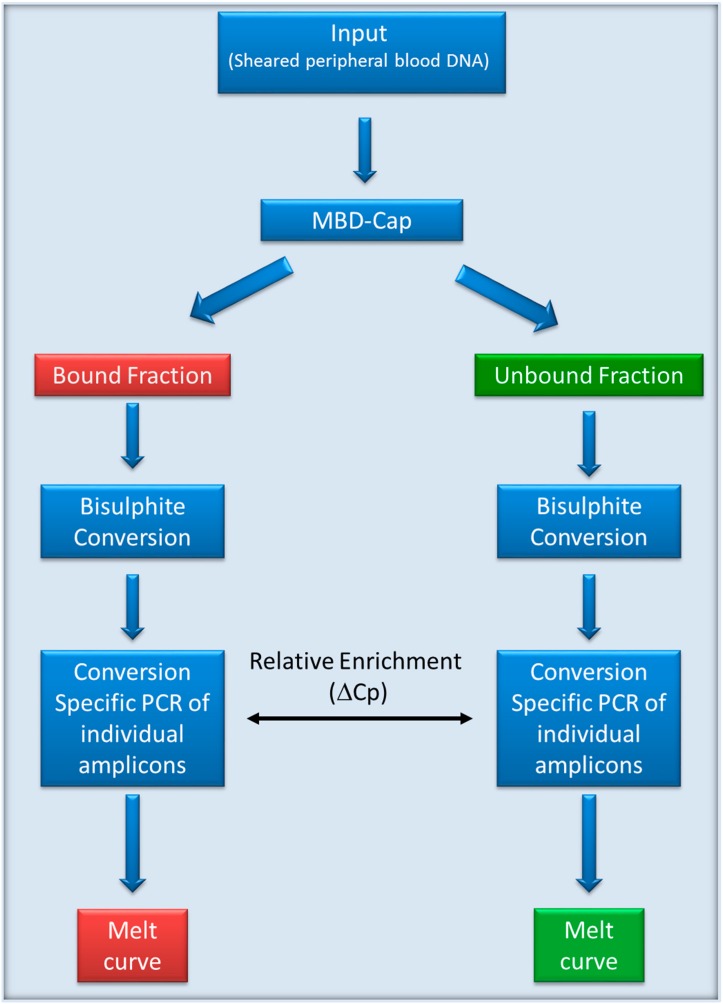
Flowchart for evaluation of methylation in white blood cell (WBC) DNA. DNA was sheared and the methylated fraction was isolated using methylated DNA binding domain-capture (MBD-Cap). The captured DNA and the unbound fraction were separately bisulphite converted. Separate conversion-specific PCRs for each gene were performed to amplify both methylated and unmethylated sequences from each of the bound and unbound fractions.

For the sensitive detection of a low level of methylated sequences in WBC DNA we adopted the protocol shown in Figure 1.

**Figure 2:**
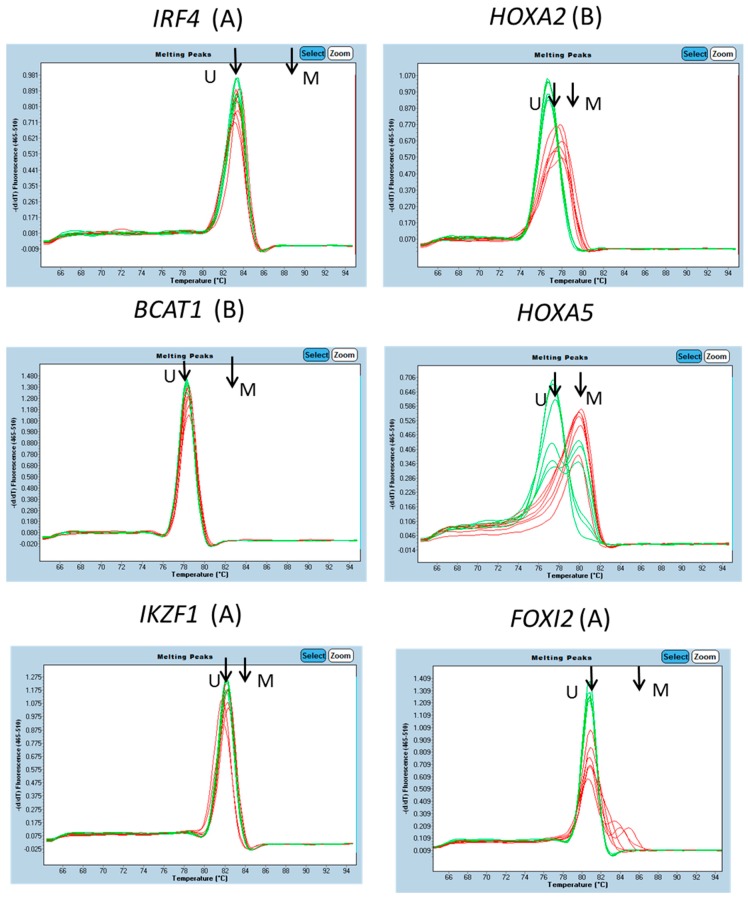
Amplicon melt curve analysis. For each amplicon, melt curves of triplicate amplifications of pooled male and female DNA (six samples in all) from the DNA fraction captured by MBD-Cap (red traces) and DNA not captured (green traces) are shown. Arrows indicated the melt peak positions for control unmethylated (U) and fully methylated (M) DNAs.

The degree of enrichment (or lack thereof) of methylated DNA sequences was estimated by comparison of the Cp values for amplification from the bound and unbound fractions. Amplicon 1 of *HOXA5* was included as a control, and the presence of significant methylation in WBC DNA had been shown. For *HOXA5*, amplification from the bound (methylated) DNA fraction occurred with a Cp slightly ahead of the unbound fraction (Table 1). This indicates that most sequences of this *HOXA5* region had been captured in the methylated fraction. Melt curves confirmed that the captured DNA was methylated and the most of the non-captured DNA unmethylated (Figure 2).

For a number of amplicons, amplification from the bound fraction occurred with a substantial delay relative to that from the unbound fraction (Table 1), a delay of about 4 to 7 cycles indicating 16 to 120-fold depletion. For example, the Cp for amplification of *IKZF1* amplicon C in the bound fraction was 37.68, while that for the unbound fraction was 31.17. This difference of 6.51 cycles indicates that the *IKZF1* DNA captured in the bound fraction was about 1/100 that in the unbound. Melt curve analyses of the amplified DNA were further used to characterize the methylation status of target regions. Compared with methylated and unmethylated controls, *IKZF1* amplicon C DNA amplified from the bound fraction was identical to that of unmethylated DNA, indicating that the low-level amplification was likely due to background non-specific capture of DNA in the methylated DNA binding domain-capture (MBD-Cap) protocol. In other cases, however, the captured DNA showed clear evidence of containing methylated sequences. For example, the MBD-Cap bound fraction of *FOXI2* region B shows a clear mix with peaks representing unmethylated sequences and methylated sequences (Figure 2). For *HOXA2* region B, the bound fraction shows a melt curve indicative of a mix of fully methylated and partially methylated molecules (Figure 2).

Based on melt curve analysis, amplicons from *BCAT1* (B), *IKZF1* (A, B & C), *IRF4* (A, B & D) (Figure 2) and *GRASP* showed no evidence of methylated DNA sequences in the MBD-Cap bound fractions, and *PDX1* a low level of partial methylation within the female DNA only. The data indicated a background binding of about 1% of input DNA in the bound fraction independent of methylation status. For other genes, *FGF5*, *SDC2* and *SOX21*, there was evidence of partial methylation but not of fully methylated molecules. Based on Cp difference and melt curve analysis regions from *EFEMP*, *FOXI2*, *GATA2*, *HOXA2*, *ZNF471* and *ZSCAN* were not considered suitable for further exploration as blood-based diagnostic markers. In considering these results it needs to be remembered that capture of DNA is dependent on the presence of more than one methylated CpG site and so is influenced by both the density of methylation and the frequency of CpG sites within the captured (sonicated DNA) fragments prior to bisulphite treatment and fragment PCR.

### 3.2. Evaluation of Selected Candidates in Human Plasma

Based on their level of enrichment in the unbound (unmethylated) fraction of WBC DNA, lack of methylation or very low level methylation evident from the melt curves of the captured DNA, and their frequency of methylation in primary CRC tissue DNA, a set of methylation targets was chosen for analysis in plasma (Table 2). Methylation specific PCR (MSP) assays to regions of interest were developed (Table S2) and used to test ccfDNA isolated from plasma obtained from colonoscopy-classified subjects (Table 2); ccfDNA from 500 µL plasma was used per triplicate assay. Data for *BCAT1* and *IKZF1* cancer and normal subjects have been published elsewhere [26].

Among the nine markers analyzed, seven showed good sensitivity (54.5%–85%) for cancer detection (*BCAT1*, *GRASP IKZF1*, *IRF4*, *SDC2*, *SOX21* and *SEPT9*), while *BCAT1*, *GRASP*, *IKZF1*, *IRF4* and *SEPT9* also showed false positive rates of ≤10%.

## 4. Discussion

Screening of ccfDNA in plasma was initially applied in the context of identifying ctDNA using specific mutations or DNA methylation markers. More recently the use of DNA methylation biomarkers has been extended to other disease conditions where cell death results in release of DNA into the circulation and the tissue of origin can be determined by the use of tissue-specific DNA methylation markers [23,24,25]. In either context, it is important to minimize DNA methylation signals arising from the major sources of ccfDNA in healthy individuals. In this context we have focused here on identifying potential background arising from WBC DNA that accounts for about 80% of ccfDNA in healthy subjects; further refinement could be obtained by applying a similar approach with normal liver DNA [23].

In the context of the detection of cancer-derived DNA, either for primary diagnosis or for monitoring of recurrence, there is a need for sensitivity for detection of a few copies of the methylated target. Given the presence of ccfDNA in the order of 10 ng per mL of plasma, even levels of methylation of less than 1:1000 in WBC DNA will contribute significantly to background. The use of MBD-Cap provides about 100-fold enrichment of methylated target sequences; with melt curve analysis we can reliably see a peak of methylated DNA corresponding to about 5% of the captured target. Thus, we expect to detect methylated sequences present at 0.1% or less for a target in WBC DNA, and use this as an exclusion criteria in prioritizing markers. Notably the two genes showing the highest level of false positives in plasma, *SOX21* and *PDX1*, both showed low levels of methylation in the MBD-Cap fraction of WBC DNA, pointing to the critical importance of extremely low background levels in WBC DNA.

Recently, bisulfite sequencing of ccfDNA, either whole genome or targeted amplicons, has been applied for analysis of plasma DNA of healthy and diseased subjects [23,24]. Provided a sufficient proportion (at least a few percent) of the ccfDNA is derived from the diseased tissue, combinations of DNA methylation, mutation and copy number information derived from the bisulfite sequencing show diagnostic promise in identifying the source tissue. There is also an increasing amount of whole-genome methylation sequence data of normal blood cell types [32,33] and other tissues [34] becoming available that will enable bioinformatic screening to eliminate certain regions as targets. The sequencing depth of available data is still limiting and measurement of low levels of methylation is also limited by the inherent background of non-conversion in the bisulfite reaction (0.1% to 0.5%). Considerable advantage could be obtained by combining MBD-Cap either with targeted (amplicon) or whole genome bisulfite sequencing of WBC DNA or of ccfDNA of healthy subjects to identify regions with the lowest levels of background.

Here we have combined MBD-Cap enrichment of methylated DNA with qPCR and melt curve analysis in order to prioritize markers initially identified from analysis of CRC tissue for clinical evaluation in plasma. Using WBC DNA pools from cohorts of nine female and nine male healthy subjects, combined quantitative and qualitative analysis of 30 genomic regions resulted in selection of nine regions for testing as prospective plasma-based biomarkers for monitoring of colorectal cancer-derived DNA; *SEPT9* was included as a control [12]. We should note that the use of WBC DNA from young adults may underestimate the likely background of individual targets if their methylation levels in WBC DNA increase significantly with normal aging. Of the genes tested in plasma, *BCAT1*, *GRASP*, *IKZF1*, *IRF4*, *SDC2*, *SEPT9* and *SOX21* exhibited high sensitivity (≈55% or greater) for detection of methylated ctDNA in cancer patients. While there was also a tendency for higher positivity rates in adenomas, the differences were not sufficient to be clinically useful. Detection of Ct DNA in plasma has been shown to depend on the invasiveness of tumors [35], and lack of sensitivity for detection of non-invasive adenomas most likely reflects a biological limitation in the capacity of blood-based tests.

The *SDC2* gene has been reported by Oh et al. [36] to show both high sensitivity and specificity for detection of cancer DNA in plasma in a Korean population study. The region used in our assay is located about 460 bp downstream in the same CpG island; the much lower sensitivity in our study sample may be due to the amplicon location or the study population. Among genes that showed good sensitivity for detection of ctDNA, *FGF5* and *SDC2* regions showed background detection in >10% of normal subjects and were not considered suitable for further blood assay development. The *SEPT9* gene gave a background of 4.5% within our subject set. Based on their low background in plasma of normal subjects, 3.5%, 6.8%, 4.9% and 4.2% respectively, the four genes *BCAT1*, *GRASP*, *IKZF1* and *IRF4* were considered suitable for assay development and optimization. Xue et al. [37] have recently reviewed studies evaluating blood-based detection of DNA methylation biomarkers for colorectal cancer and the above represent promising biomarkers that could be combined with these or other markers to potentially provide improved sensitivity for cancer detection. In combining biomarkers to improve assay sensitivity, it is critical that individual markers have minimal false positive detection rates in normal subjects in order to maintain good specificity. In a 218-person case/control study of two of our prospective biomarkers, *BCAT1* and *IKZF1*, the combination of two markers provided greater sensitivity (77%) compared to the two individual assays (65% and 67%) but at the cost of a higher false positive rate (7.6%) compared to the individual markers (3.5% and 4.9%) [28]. This combination has now been evaluated in two prospective clinical trials including more than 3900 subjects [29,30] and showed an overall sensitivity and specificity of 66% and 94% respectively for detection of colorectal cancer [30].

These data demonstrate the utility of close consideration of the background levels of DNA methylation in WBC DNA as an important step in the selection of biomarkers suitable for development as plasma-based assays.

## Figures and Tables

**Table 1:**
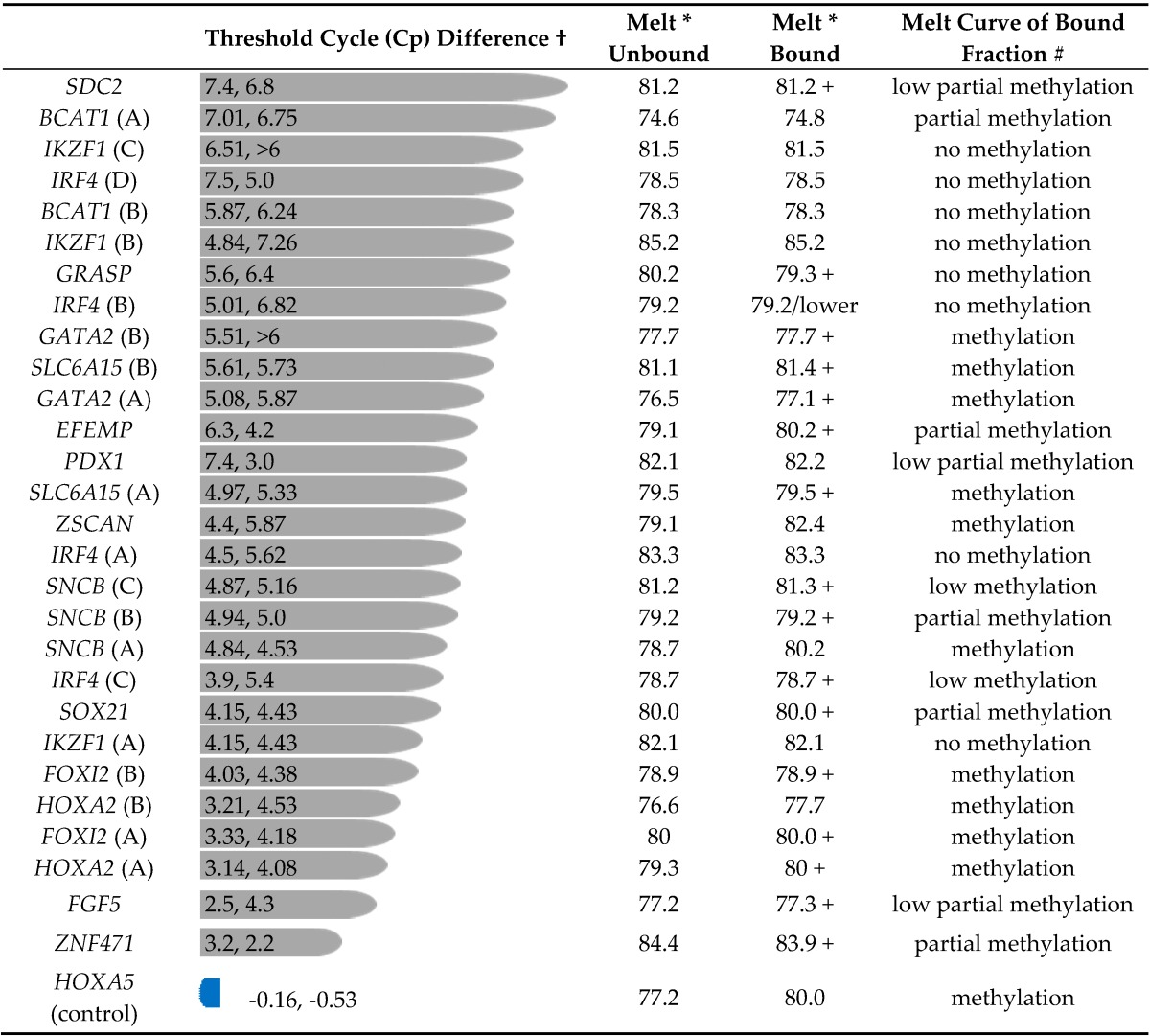
Amplification from MethylMiner captured and unbound fractions.

**†** Difference between the Cp values for amplification from bound fraction (methylated DNA) and unbound fraction for male and female DNAs respectively; bars length scaled to average Cp difference. Table ordered from top by Cp difference; * T_m_ of main peak in melting profile of amplified DNA; + indicates that there was also material with a higher melting temperature than the main peak; # Partial methylation: contains amplified material with melting temperature in between that of fully methylated and fully unmethylated DNA Methylation or low methylation: contains (some) amplified material with melting temperature equivalent to that of fully methylated DNA.

**Table 2:** Frequencies of detection of methylated DNA using methylation-specific PCR assays.

	Cancer		Adenoma		Normal	
	Number Tested	Number (%) Positive	Number Tested	Number (%) Positive	Number Tested	Number (%) Positive
***BCAT1***	74	48 (64.9)	33	4 (12.1)	144	5 (3.5)
***FGF5***	20	17 (85)	40	13 (32.5)	40	7 (17.5)
***GRASP***	44	24 (54.5)	44	6 (13.6)	44	3 (6.8)
***IKZF1***	74	50 (67.6)	33	8 (24.2)	144	7 (4.9)
***IRF4***	22	13 (59.1)	21	2 (9.5)	24	1 (4.2)
***PDX1***	20	9 (45)	20	4 (20)	20	6 (30)
***SDC2***	44	26 (59.1)	44	6 (13.6)	44	7 (15.9)
***SEPT9***	44	26 (59.1)	44	2 (4.5)	44	2 (4.5)
***SOX21***	20	17 (85)	17	9 (52.9)	20	10 (50)

## Supplementary Materials

The following are available online at www.mdpi.com/2073-4425/7/12/125/s1, Figure S1: Methylation in cancer and normal subjects: TCGA array probe data, Table S1: Oligonucleotide primers for conversion specific PCR assays, Table S2: Primers, probes and amplification conditions for methylation specific PCRs, and Table S3: Plasma assay triplicate data.
