# Supplementary material for: Evaluation of Methylation Biomarkers for Detection of Circulating Tumor DNA and Application to Colorectal Cancer
Source: Genes (Basel). 2016 Dec 15;7(12):125. doi: 10.3390/genes7120125 (PMC5192501; doi:10.3390/genes7120125)
Supplement: Supplementary file 1 [file genes-07-00125-s001.zip › genes-147510-supplementary.pdf]

Genes **2016**, *7*, 125; doi:10.3390/genes7120125

Supplementary Materials: Evaluation of Methylation Biomarkers for Detection of Circulating Tumor DNA with Application to Colorectal Cancer

Susan M. Mitchell, Thu Ho, Glenn S. Brown, Rohan T. Baker, Melissa L. Thomas, Aidan McEvoy, Zheng-Zhou Xu, Jason P. Ross, Trevor J. Lockett, Graeme P. Young, Lawrence C. LaPointe, Susanne K. Pedersen and Peter L. Molloy

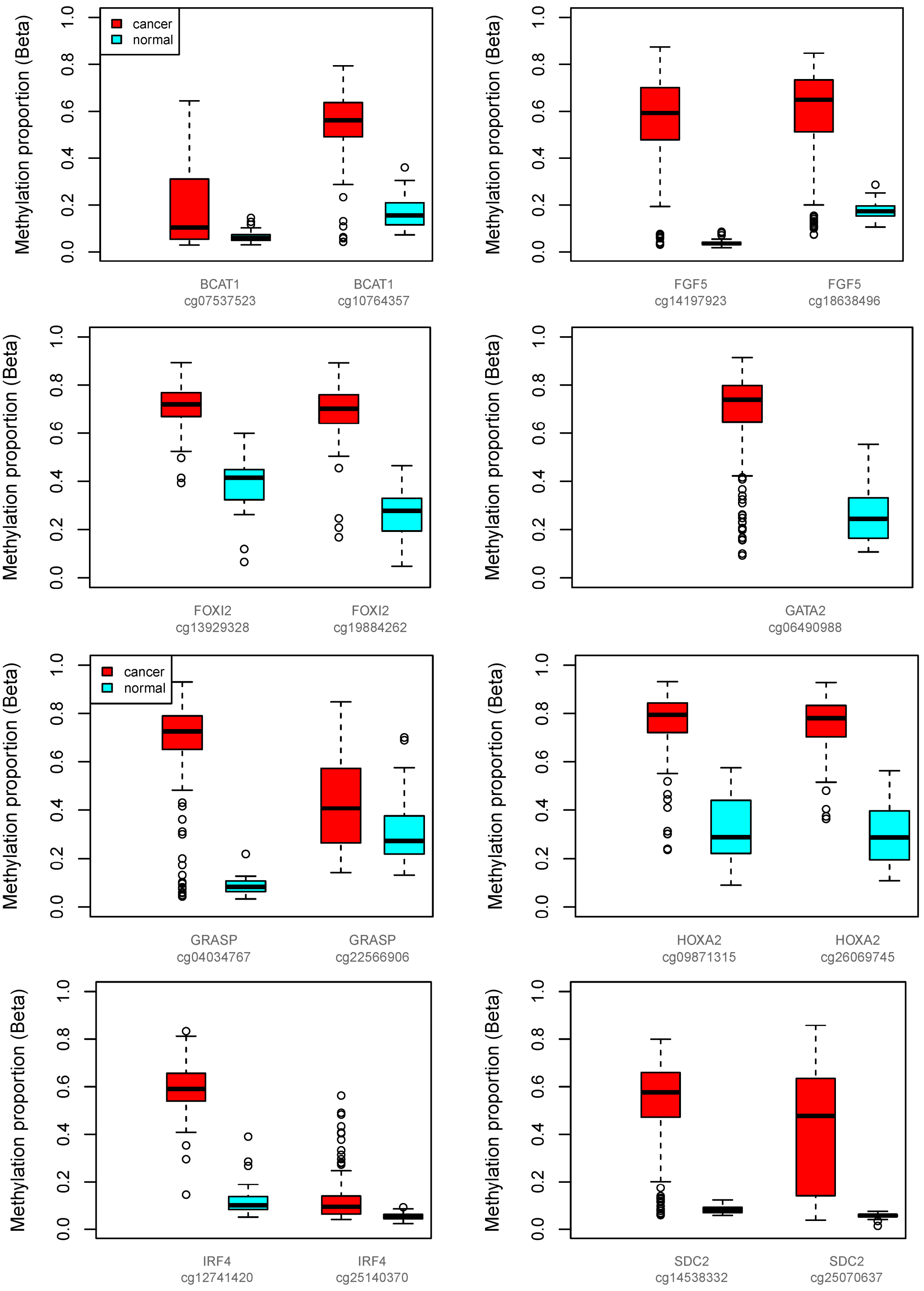

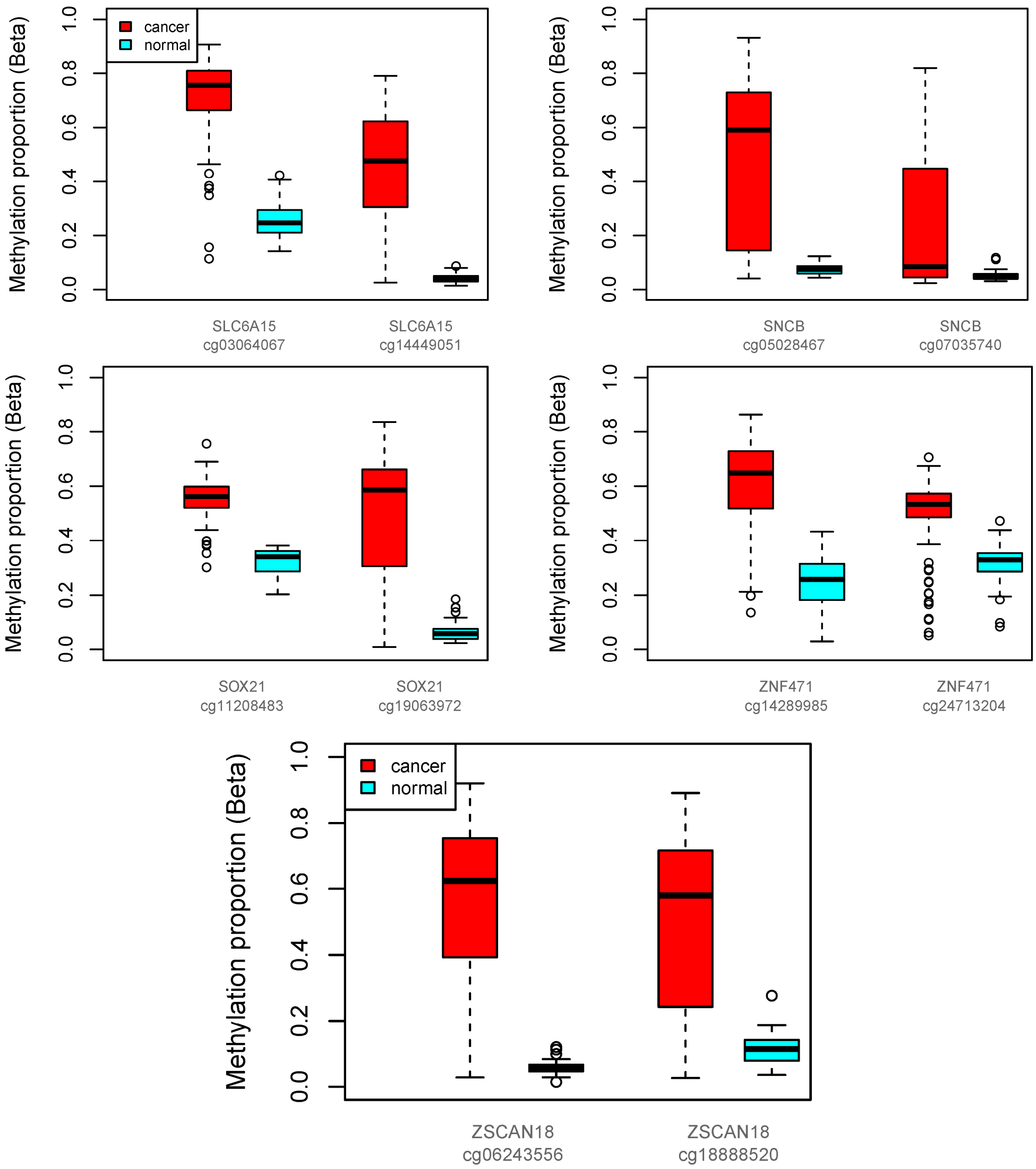

Figure S1. Methylation in cancer and normal subjects: TCGA array probe data. Box plots show ranges of methylation at individual probes on the Illumina Infinium Human Methylation27K bead arrray data from the Cancer Genome Atlas consortium. Red: colorectal cancer tissue samples, Blue: normal colorectal tissue. Plots are included for all genes where probes were within 800 bp of target regions and were used to guide design of conversion-specific amplicons.
